# Studying Chinese Foreign Policy Narratives: Introducing the Ministry of Foreign Affairs Press Conferences Corpus

**DOI:** 10.1007/s11366-021-09762-3

**Published:** 2021-09-23

**Authors:** Michal Mochtak, Richard Q. Turcsanyi

**Affiliations:** 1 Institute of Political Science, Maison des Sciences Humaines, University of Luxembourg, 11 Porte des Sciences, L-4366 Esch-sur-Alzette, Luxembourg; 2 Department of Asian Studies, Palacky University Olomouc, Křížkovského 511/8, 779 00 Olomouc, Czech Republic

**Keywords:** Corpus, Foreign affairs, Press conferences, Narratives, Chinese foreign policy

## Abstract

The paper presents an original corpus of the Chinese Ministry of Foreign Affairs press conferences. The dataset is a unique source of information on official positions and diplomatic narratives of China mapping almost two decades of its foreign policy discourse. The corpus contains almost 23,000 question – answer dyads from 2002 to 2020 ready to be used for analytical purposes. We argue the dataset is an important contribution to the scholarship on Chinese foreign policy stimulating further research using corpus based methods while employing both qualitative and quantitative strategies. We demonstrate possible applications of the corpus with two case studies: first maps the diplomatic discourse towards the US under the presidency of Hu Jintao and Xi Jinping (employing quantitative tools), while second analyzes narratives concerning the South China Sea disputes (employing more qualitative approach).

## Introduction

Studying politics would be impossible without acknowledging that language and politics are intimately linked at a fundamental level. In fact, political activity cannot exist without the use of language formalized as narratives. Although other activities co-create politics and define its complex multidimensional structure, the communicative role of language appears to be essential for its content. Narratives in this context tell us ‘who we are’ (identity) and ‘what we want’ (interests) [5]. This is especially true when it comes to states as actors of international relations constructing social reality in order to configure their preferences, identities, and social reality [16]. In fact, strategic narratives have potential of changing the international reality in a significant way and it was argued that they are one of essential prerequisites of an emerging great power [31].

Chinese politics is notoriously infamous for its opaque nature with generations of experts struggling to understand what is going on behind the closed doors of Zhongnanhai. While existing literature has increasingly recognized that, in this context, narratives play a crucial role in understanding China’s political behaviour [21, 43], complex and reliable resources that would explicitly identify how narratives emerge, develop, and become dominant in Chinese foreign policy discourse remain scarce [64].

To address this gap, the paper introduces an original corpus of Chinese Ministry of Foreign Affairs Press Conferences^1^ which maps almost two decades of Chinese diplomatic discourse (2002–2020). The Chinese Ministry of Foreign Affairs (the Ministry from now on) have organized regular press conferences for decades representing an authoritative voice of the regime in foreign affairs [51]. Interestingly, although commentaries presented at the Ministry’s regular press conference are well known and are often referenced by scholars, they have been rarely used beyond a supporting anecdotal evidence. One reason may be simply due to the fact that the ministry’s webpage shows only transcripts from the previous two years and the webpage interface is not easy to navigate and locate the desired information. We have, however, managed to gather data from the press conferences since 2002 and have compiled them systematically in a single structured dataset ready to be used for various analytical purposes. We argue that this dataset can be an important contribution for future research of Chinese foreign policy employing both qualitative and quantitative methods.

To present the dataset, the paper demonstrates two applications focused on highlighting changes in Chinese foreign policy narratives. First, employing an innovative quantitative approach, we visualize and interpret transformations of foreign policy discourse related to the US between the Hu and Xi eras, which has been so far addressed using mainly qualitative approaches combined with interpretative methods. Second, we use more qualitative approach to track Chinese official narratives in relation to the South China Sea disputes, and particularly towards various involved claimant and non-claimant actors. These two case studies are not necessarily meant to discover new findings. We have selected these often-researched areas of China’s international relations deliberately, first, to demonstrate the potential of dataset to a broad research community and second, to validate its merit and relevance when it comes to China’s official narratives. As such, the paper presents the case studies as a proof of concept when it comes to corpus based methods and their application in the context of China’s foreign policy discourse. The reasoning for validation follows an assumption that if the cases could not be used for analyzing well-accepted changes in China’s foreign policy, the corpus would be much less valuable to scholars studying China while its overall integrity could be easily questioned. The results, however, show that the corpus does indeed yield the descriptive inferences we would expect. The case studies should give readers confidence that future applications of the corpus to new (and less understood) aspects of Chinese foreign policy will provide equally valid measurements.

### China’s Official Discourse: From the “Low Profile” Diplomacy to “Chinese Assertiveness”

Around 2010, scholars studying China started pointing out that the course of Chinese foreign policy has been altering and China had become more “assertive” [44]. For about twenty years, Chinese government had mostly followed the dictum introduced by former paramount leader Deng Xiaoping on practicing a “low profile diplomacy” [10]. However, various incidents in late 2000s seemed to suggest that the era was coming to its end with the new approach being labelled as “assertive” [17, 19]. Although evidence was initially inconclusive [12, 23, 25], shortly after Xi Jinping took over from Hu Jintao in 2012, it became clear that the new leader would make his growing power and influence felt domestically as well as internationally [60], in many respects abandoning the trajectories Deng Xiaoping introduced in late 1970s [42].

Although “Chinese assertiveness” was perceived (predominantly in the West) as inherently “anti-Western” from the beginning [49, 50], it was primarily directed toward Chinese geopolitical neighbourhood and disputed territories, such as the South China Sea [59, 65]. At about the same time, David Shambaugh [44] still refrained from exaggerating China’s global influence calling it at most a “partial power” which has only limited presence in many geographical and issue areas of global politics. The following China-meme of “Chinese influence”, however, expended to a global prominence and reached ‘peripheral’ developing world [33, 46] as well as the ‘core’ of developed countries [6, 55]. In this awakening, China came to be seen as the main challenger of the Western-led international order at every possible front [35].

In the rapidly polarizing environment with growing stakes, research of China-related issues faces challenges of increasing politicization. One way how academics try to deal with the new status quo has been the focus on discourse. Recent studies have mapped the patterns of Western narratives towards China arguing that some popular arguments may have questionable empirical basis and lead to ineffective policy responses, possibly even creating self-fulfilled prophecies [7, 39, 52]. More effort is also seen in the study of China’s political discourse with the focus on topics like political slogans [26], narratives in Chinese foreign policy [22, 37, 47], overlap of international and domestic issues [30, 56], uniqueness of Chinese discourse in general [45], personal attacks used by the spokespersons [36], politicians’ and journalists’ aggressiveness [58], or construction of national face [29].

Recently, China’s official discourse has become an increasingly attractive area of study for scholars, spurred by developments such as Xi Jinping’s ascension to power, Belt and Road Initiative, and eventually Covid-19 and growing tensions between China and the West [22]. Lutgard Lams [27], Yi Edward Yang [61], and Kerry Brown [8] discussed development of Chinese strategic narratives under Xi Jinping, compared to the previous eras, specifically focusing on international and domestic audiences and identifying dominant frames. Indeed, the interplay between the domestic and international contexts appears prominently in the studies of China’s official discourse and has been addressed extensively by Kingsley Edney in his study of propaganda [15]. Yifan Yang and Xuechen Chen [62] focused on a similar aspect within China’s official discourse: the interplay of globalist and nationalist language during the Covid-19 pandemic. Yung-Yung Chang [9] focused on how Covid-19 pandemic would impact China’s narratives at the three levels (international system, national, and issue narratives) in terms of their formation, projection, and reception.

Finally, when discussing China’s official narratives, one must take note of the Belt and Road Initiative (BRI), which has arguably worked to a great extent with strategic narratives as a policy tool, and it has been studied by scholars from these perspectives. In fact, proponents of the study of strategic narratives Miskimmon, O’Loughlin, and Zeng have devoted an edited volume to the particular issue of BRI and EU-China strategic narratives [32]. Elsewhere, Lina Benabdallah argued that the selection of the BRI narratives employs ‘nostalgia’ for political purposes [4], while other works have focused on how BRI and related narratives have shaped European receptions [28, 53, 54].

Despite a growing body of literature, there has been relatively little effort to study Chinese foreign policy discourse by employing more rigorous data, quantitative approaches, and specifically using computational methods. We argue that our dataset of almost twenty years of the Ministry of Foreign Affairs press conferences is an important contribution to the scholarship on Chinese foreign policy with a potential use within both qualitative and quantitative traditions of political science, international relations, economy, and other fields studying contemporary China and its interactions with the world.

### Introducing the Chinese Ministry of Foreign Affairs Press Conferences Corpus

Regular press conferences organized by Chinese Ministry of Foreign Affairs are unique source of information on official positions taken by Chinese government on different topics in international relations. The press conferences follow a western style structure with journalists asking questions and spokespersons responding to them. Although journalists are generally free to ask questions, hence acting as potential agenda-setters [34], the answers are provided selectively from a position of authoritative superiority creating an echo chamber for Chinese foreign policy. In other words, even if journalists might ask all sorts of questions, whether they eventually make it to the official transcript depends solely on the Ministry. Moreover, the transcripts of press conferences are curated skewing the overall influence of journalists (and partially also individual Ministry’s spokespersons) on the actual message the Ministry wants to disseminate [14, 63]. It should be also emphasized that the published transcripts are highly scripted, with possible sections omitted or adjusted as a result of standardization or outright censorship. In other words, the Corpus should not be regarded as exact transcriptions of what was actually said at the conference – instead it is what the Ministry wants to be publicly heard [63].

For the purposes of the study of China’s official discourse, however, this is perfectly acceptable – in fact, for the very same reasons, the published texts in English on the Ministry’s website can be regarded as highly authoritative official positions of the Chinese state on given issues in the way it wants them to be communicated to the world. As such, the Corpus should not be treated primarily as the representation of Chinese leaders’ intentions and beliefs – these may, naturally, remain hidden. However, it is still a unique source of Chinese strategic narratives used as a diplomatic tool meant to attract, persuade, threaten, and otherwise achieve broader goals of Chinese foreign policy which requires interaction with foreign actors. We have thus decided to focus on the English-language discourse (instead of working with Chinese-language discourse) as this can be regarded as being intended for the broadest international audience (all available languages concerned).^2^ It might be nonetheless worthwhile to conduct similar exercise for Chinese language transcripts, and potentially other languages, in future, and compare the results.^3^

Unfortunately, the Ministry’s website is programmed in such a way that only approximately two years of transcripts are publically available at a time. As our goal was to collect as much data as possible, an alternative approach must have been applied. We have collected all publicly available transcripts of press conferences from the official website in three stages (30 September 2018; 30 September 2019; 25 March 2021) using crawlers programmed with *rvest* and *RSelenium* in R [18, 57]. That gave us approximately four years of data mapping the Chinese foreign policy discourse. For older dates, we used the Wayback Machine platform operated by Internet Archive, which has been archiving internet websites since 1996. Today the Internet Archive has over 20 years of web history accessible through the Wayback Machine API engine [2]. We used the *wayback* package to access the archive’s API and extract whatever information collected over the archived period [40]. This approach allowed us to go back as far as October 15, 2002 when the first working snapshot of the Ministry’s press conferences website allows accessing that date. Although there is no guarantee we have acquired all transcripts of press conferences ever published by the Ministry, the density of snapshots available via Archive’s API from October 2002 makes us to believe the actual coverage is close to 100%.^4^

**Fig. 1:**
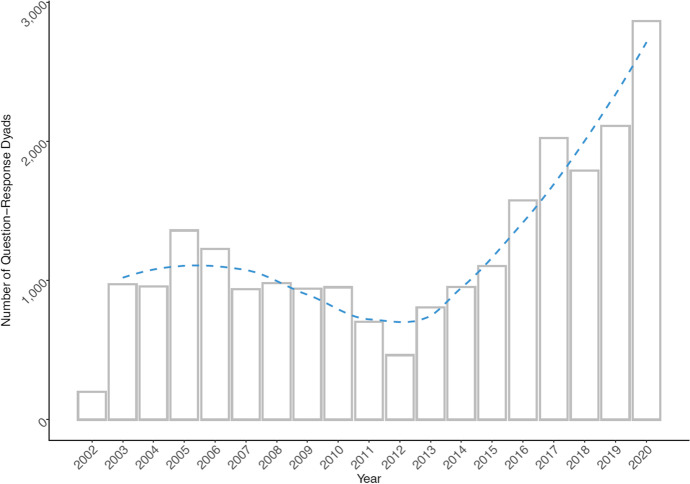
Distribution of question-response dyads over time. Note: Dashed line visualizes smoothed trend for the whole years (using loess function [Local Polynomial Regression Fitting])

**Fig. 2:**
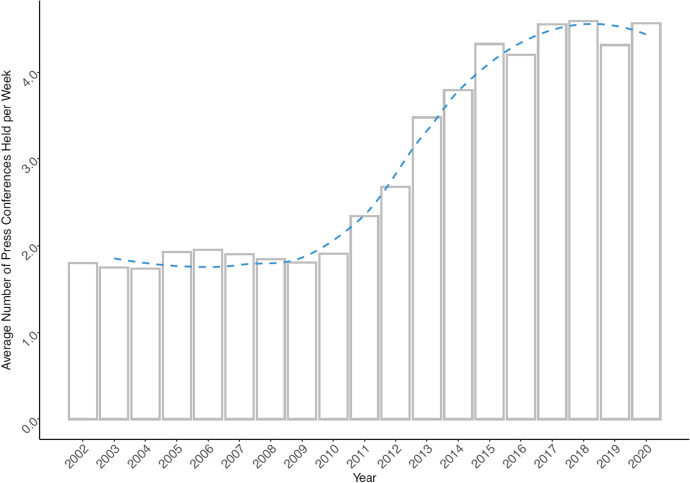
Average number of press conferences held per week. Note: Dashed line visualizes smoothed trend for the whole years (using loess function [Local Polynomial Regression Fitting])

**Fig. 3:**
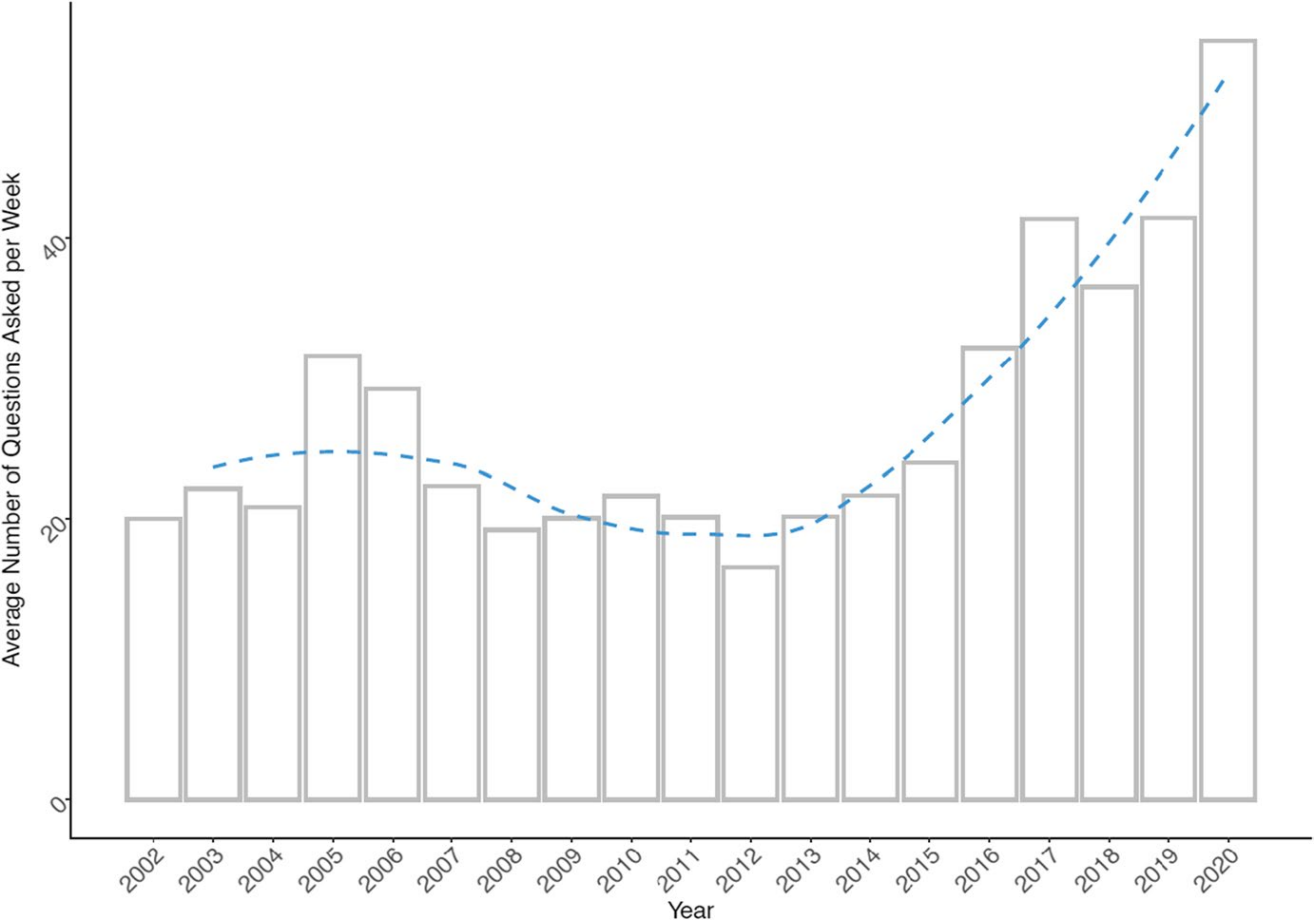
Average number of questions asked per week. Note: Dashed line visualizes smoothed trend for the whole years (using loess function [Local Polynomial Regression Fitting])

With raw textual data at hand, we developed a series of cleaning and pre-processing routines for detection of different styles in transcripts. The goal was to extract the question/response structure of transcripts and accompany them by a proper timestamp as well as information on which spokesperson held the press conference.^5^ The question–response dyad comprise what is called an “adjacency pair” in conversation analysis considered to be the fundamental unit for conversation organization [41]. The proto corpus created this way covers period from 15 October 2002 to 31 December 2020 and in its raw form counts 21,355 question–response dyads. A dyad refers to a pair of a question asked by a journalist and a response provided by the spokesperson. As journalists may ask multiple questions often covering various topics, we decided to split questions having a clear multi-topic character into separate entries using rule-based algorithms and manual coding (new and old IDs are preserved separately). As a result, the total number of single-topic dyads increases to 22,946 (see Fig. 1 for their frequency distribution over time). In its raw form, the corpus has 874,051 types (total words) and 19,787 tokens (unique words) in its question part and 2,752,739 types and 25,760 tokens in its response part. The obvious disproportionality between the Hu Jintao era and Xi Jinping era (especially considering the recent years) is a result of transformation of the role press conferences have in the system of MFA’s public relations [14, 63]. Figure 2 visualizes the change of average number of press conferences per week from 2002 to 2020 and shows a gradual increase of average number of press conferences form two to over four in the post-2011 period (Xi Jinping era). Figure 3 shows substantially similar trend when it comes to average number of question-response dyads reported on weekly basis.

For most of the research needs, topical distribution and, more specifically, Chinese discourse towards different international actors or agenda would be helpful. As manual coding of almost 23,000 question/response dyads would require an enormous effort and labour-intensive work, we decided to apply an automated algorithm for extracting named entities, which can be assigned back to specific questions/responses as their topical labels. We use pre-trained models for extracting entities focused on locations, persons, organizations, and miscellaneous references [1]. The extracted entities can be used for assigning topical tags based on research preferences, which can range from geographical focus to geopolitical issues. Although not essential for using the dataset, the pre-processed tags assigned to individual dyads allow us to split the corpus into topics that can be further analysed. To streamline the research process for researchers interested in Chinese discourse in general, we populated the corpus with lemmatized version of recorded question/response dyads, which can be used for information extraction as well as summarization. We also accompany the corpus dataset with full annotations of the collected questions and responses.

### Case Study #1: Changes in China’s Diplomatic Discourse Related to the US

To demonstrate the usefulness of the dataset as well as to validate its general integrity, the first case study employs selected quantitative tools and explores the traces of Chinese meta-discourse concerning the US. The case study builds on the fact that the US has played a crucial role in China’s international relations for decades and as a leader of the liberal democratic world, it can be regarded as the “significant other” for the Communist China. Moreover, after decades of dynamic rise, by the end of 2010s (at the latest), China has established itself by most counts as the second most powerful nation-state in the international system and perhaps the only serious peer contender to the US – although still arguably from far away [52]. As we saw previously, the US has been central to both “low profile” diplomacy – when China was not interested in confrontations and focused primarily on the economy and cooperation – as well as during the periods of “Chinese assertiveness” and “Chinese influence”, when China came to be perceived as opening new fronts in anti-Western and anti-US activity. Looking at how the US appears in the Chinese official foreign policy discourse is of outmost importance both as an expression of Chinese foreign policy as well as a way to understand self-perception of Chinese government in relation to its main peer rival. The collected dataset allows us to explore both longitude dynamics, as well as topical connections between various issues.

To begin, we took the extracted named entities and analyse them for the occurrence of keywords that explicitly refer to the United States as a country or its highest representatives. This includes a narrow country references (US; USA, United States; Washington) as well as its main representatives (presidents [Bush, Obama, Trump] and secretaries of state [Pompeo, Tillerson, Kerry, Clinton, Rice, Powell]). If any of the keywords is found, the list of extracted entities, a tag referring to US, is assigned to a specific question/response dyad. We use these tags for sub-setting the corpus only to responses concerning the US so they can be analysed for prevailing Chinese discourse in relations to the country in question. In order to highlight the potential differences in prevailing narratives before and after the change in Chinese leadership (Hu Jintao [2002–2012]; Xi Jinping [2012 - present]), we split the sub-corpus to period before and after November 15, 2012 (see summary in Table 1). To map the overall discourse, we first focus on changes in sentiment polarity in answers concerning the US. Second, we summarize the changes identified under Hu Jintao and Xi Jinping as consistent phrases that are present in our datasets and visualize them in a discourse network. The imbalanced data is not an issue for any of the presented analysis as both sentiment scores and pairwise correlations of words (i.e. consistent phrases) are normalized within their respective subcorpora (average sentiment is measured on the level of sentences; probabilities of co-occurrences of words are relative to number of sentences).

**Table 1:** Summary of corpora mapping US-China discourse

Corpus	Number of Responses	Number of sentences	Number of words (after cleaning)	Number of unique words (after cleaning)
US-related discourse in the Hu Jintao era	2651	15,197	171,404	6600
US-related discourse in the Xi Jinping era	4658	33,327	404,075	10,981

**Fig. 4:**
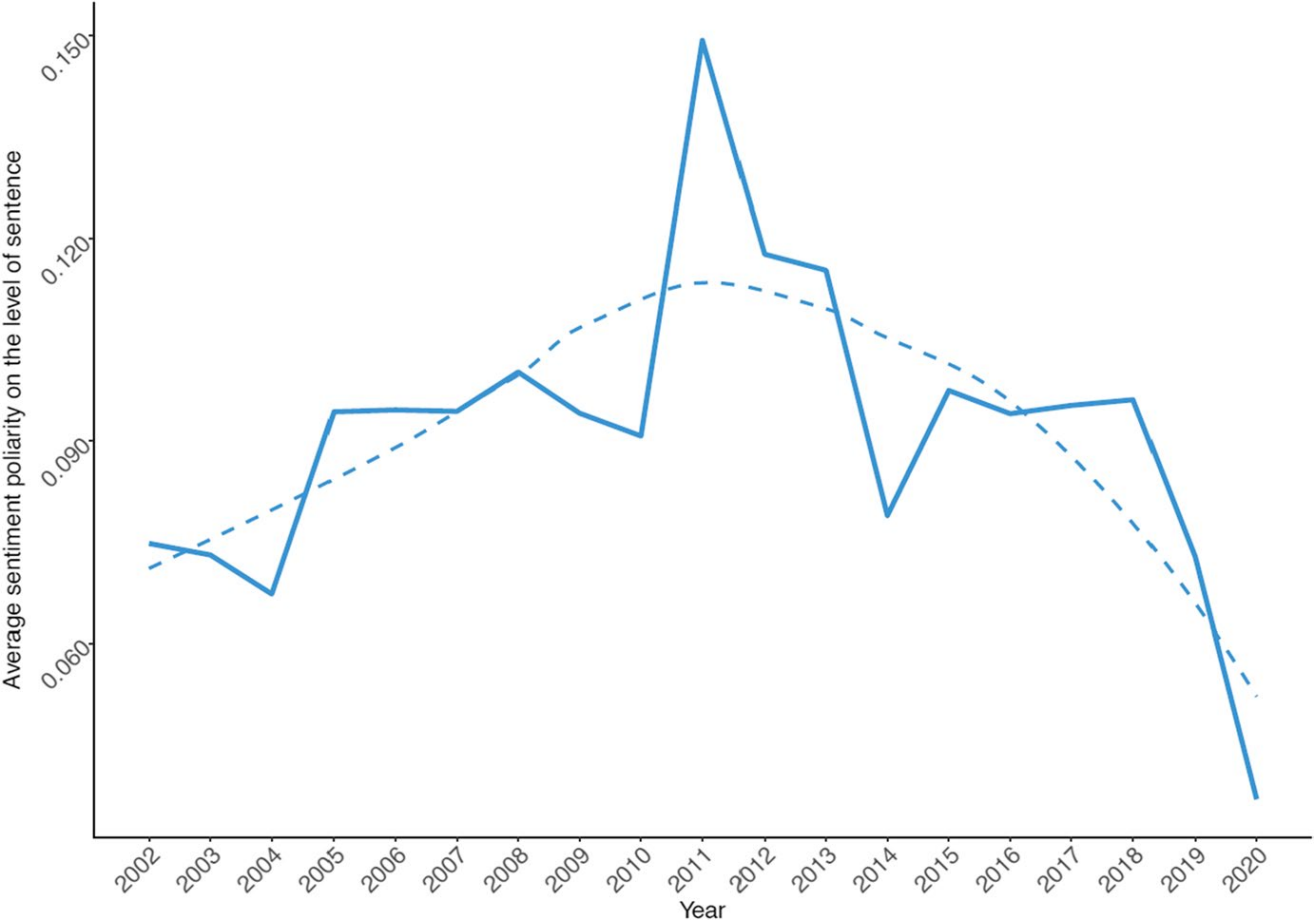
Change in sentiment polarity towards the US in Chinese foreign policy discourse. Note: Solid line visualizes raw sentiment averages on the level of sentences. Dashed line visualizes smoothed trend (using loess function [Local Polynomial Regression Fitting])

When it comes to overall sentiment towards the US, the trend visualized in Fig. 4 captures the overall relations between both countries over the past two decades. For counting sentiment, we use R package sentimentr [38] which takes into account valence shifters (i.e., negators, amplifiers, de-amplifiers, and adversative conjunctions) and utilizes them in a counting pipeline of dictionary lookups of known polarity words (in this case we use the default dataset containing a combined and augmented version of Jockers [24] and Rinker’s augmented Hu and Liu [20] positive/negative word list counting 11,710 items). We summarize the average sentiment on a sentence level on a yearly basis and normalize it on a scale from −1 (most negative) to 1 (most positive).

As we can see, the overall sentiment of Chinese official discourse involving the US over the previous two decades can be roughly divided into two eras, coinciding with the Chinese leaders Hu Jintao and Xi Jinping. Pre-2012 era has seen improving sentiment of the US, reaching its peak in 2011, the last full year of Hu’s leadership. Since then, with Xi Jinping in office, the sentiment of the discourse involving the US has been worsening, accelerating after 2018 and reaching the lowest (i.e. most negative) spectrum over the entire studied period. This finding is generally in line with our expectations based on the review of the US-China relations in the previous 20 years.

**Fig. 5:**
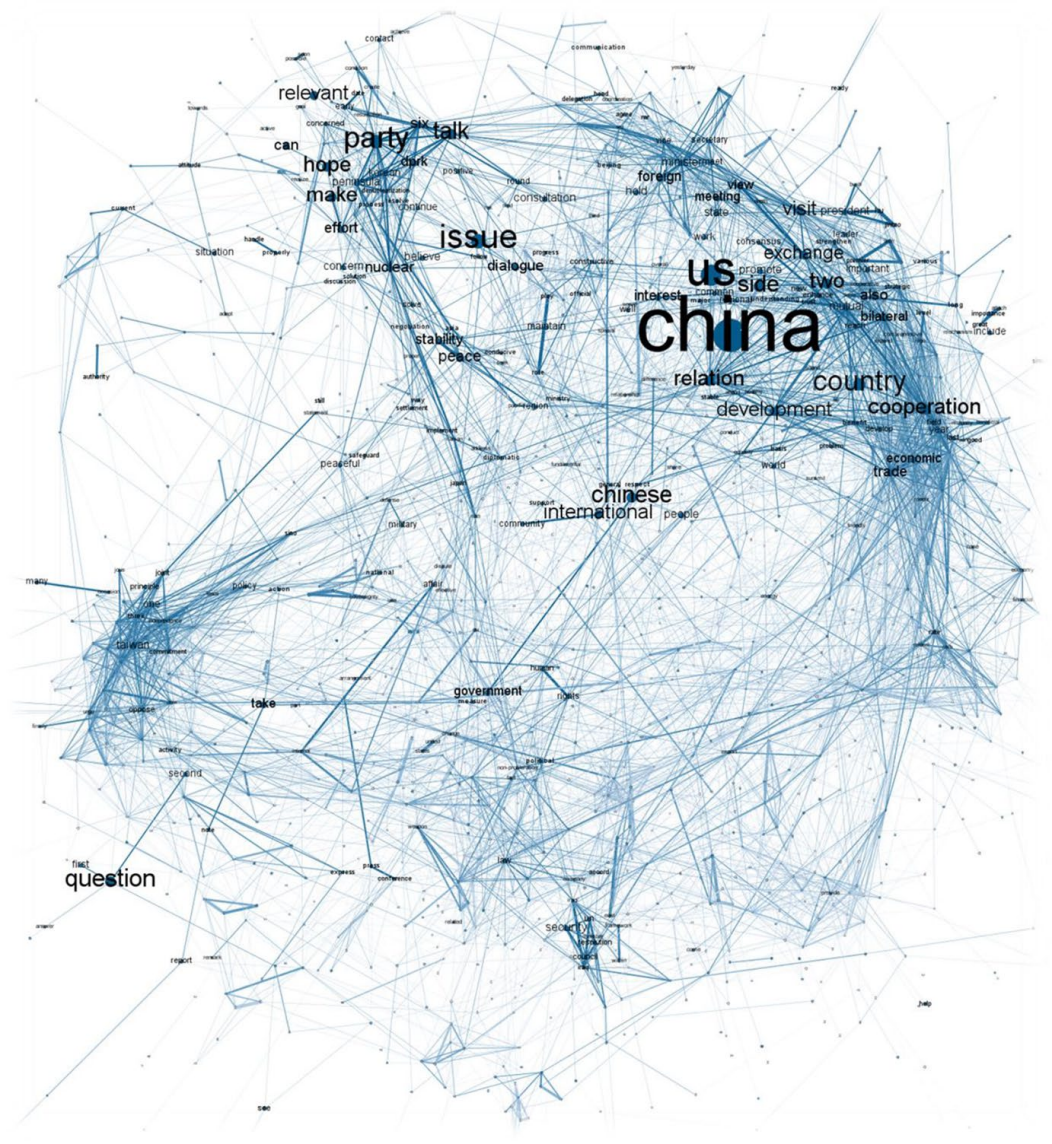
Semantic network tracing US-China discourse under President Hu Jintao

**Fig. 6:**
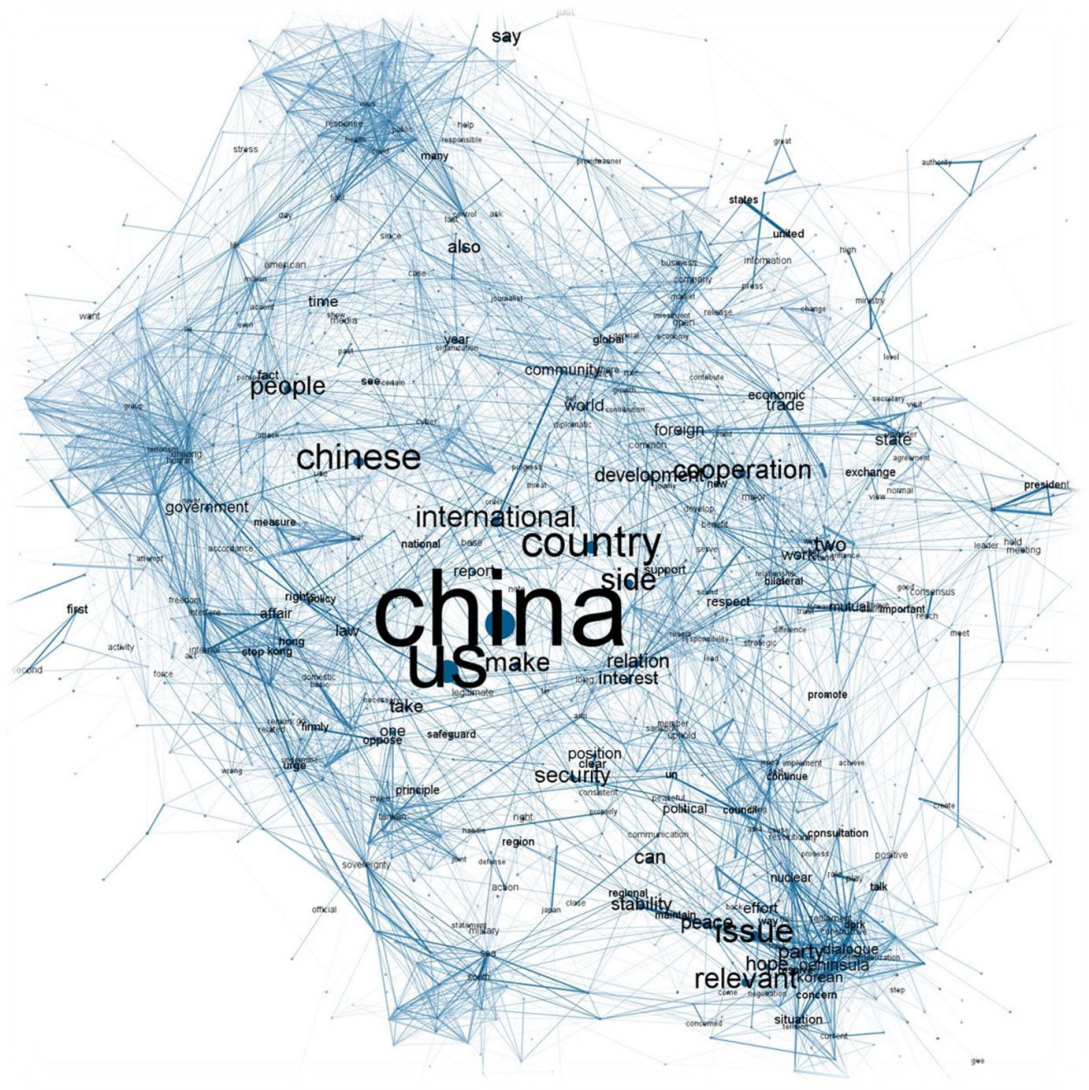
Semantic network tracing US-China discourse under President Xi Jinping

When it comes to actual discourse, each sub-corpus is analysed separately using models based on word colocations. More specifically, we build two models using pairwise correlations computed among 1500 most frequent words. In order to do that, we split the collected answers into sentences, which are used as natural units for words’ co-occurrences. The colocation analysis returns a list of pairs of co-occurring words with phi coefficients indicating empirically consistent pairs. As listing thousands of word pairs would be highly impractical, we apply a network approach in order to capture the complexity of discourse on a macro level (visualizing pairs of words with phi coefficient > = 0.15). We present our findings graphically in Figs. 5 and 6 where the size of the nodes (labels) refers to words’ document frequency, colour hue visualizes words’ absolute rank in an ordered frequency list, and edge thickness depicts consistency of how words co-occur together (phi coefficient). Both graphs show labels that are among the first 300 most frequent words in the respective sub-corpus. The visualization is done using Gephi v0.92 and Force Atlas 2 algorithm [3]. The replication code as well as the graphs in high resolution are available via Harvard Dataverse repository (see note 1).

As we can see, the Chinese diplomatic discourse concerning US has clearly changed between the two analysed period. While the pre-2012 discourse is dominated by primarily three topics – US-China bilateral relations; situation on Korean Peninsula; and Taiwan – post 2012 period introduces a number of new topics, including the events in South China Sea or Hong Kong. This roughly copies the general dynamics of US-China relations during the past two decades. In the Hu period, we can see the relationship centred around the (economic) cooperation with only occasional conflicts (such as related to Taiwan), while in Xi period more conflictual topics arise creating an overall more confrontational relationship [7, 22]. To explore this further, we focus on issues that stand in clear opposition to a cluster of US-Chinese bilateral relations framed through cooperation, development, and mutual understanding. While the pre-2012 discourse places Taiwan as the only issue truly opposing the cluster of cooperation, with the situation concerning Korean Peninsula situated somewhere in the middle, the post-2012 discourse sees multiplication of such issues potentially widening the gap between official conciliatory language focused on cooperation and topics that are more conflicting. The close proximity of clusters around Taiwan, South China Sea, and Hong Kong also shows how the Ministry frames the issues while considering the US positions – all three of them are seen as internal affairs of China with US activities being considered as unwelcomed interferences [49]. Both semantic networks also show that from China’s perspective these conflicting topics only lightly interfere with the general US-China bilateral relations, which might indicate that China develops “several foreign policies”, which do not have to interfere with each other. Again, these findings can be regarded as validating our approach as they are generally in accordance with the well-known trajectory of the US-China relations. At the same time, while not necessarily ‘surprising’, our findings already provide novel insights into the dynamics of US-China relations by offering new substantial empirical basis to describe the US-China relations more accurately.

### Case Study #2: South China Sea Disputes

To demonstrate a more qualitatively oriented approach utilizing our dataset, we now focus on disputes concerning South China Sea and the change in official China’s discourse over the years 2002–2020. South China Sea is a particularly relevant example of the topic to study changes in Chinese foreign policy during the Hu and Xi eras, as it has been recognized as one of the key areas where the change from “low profile” diplomacy towards “assertiveness” was noticed [25, compare with 12, 52].

**Fig. 7:**
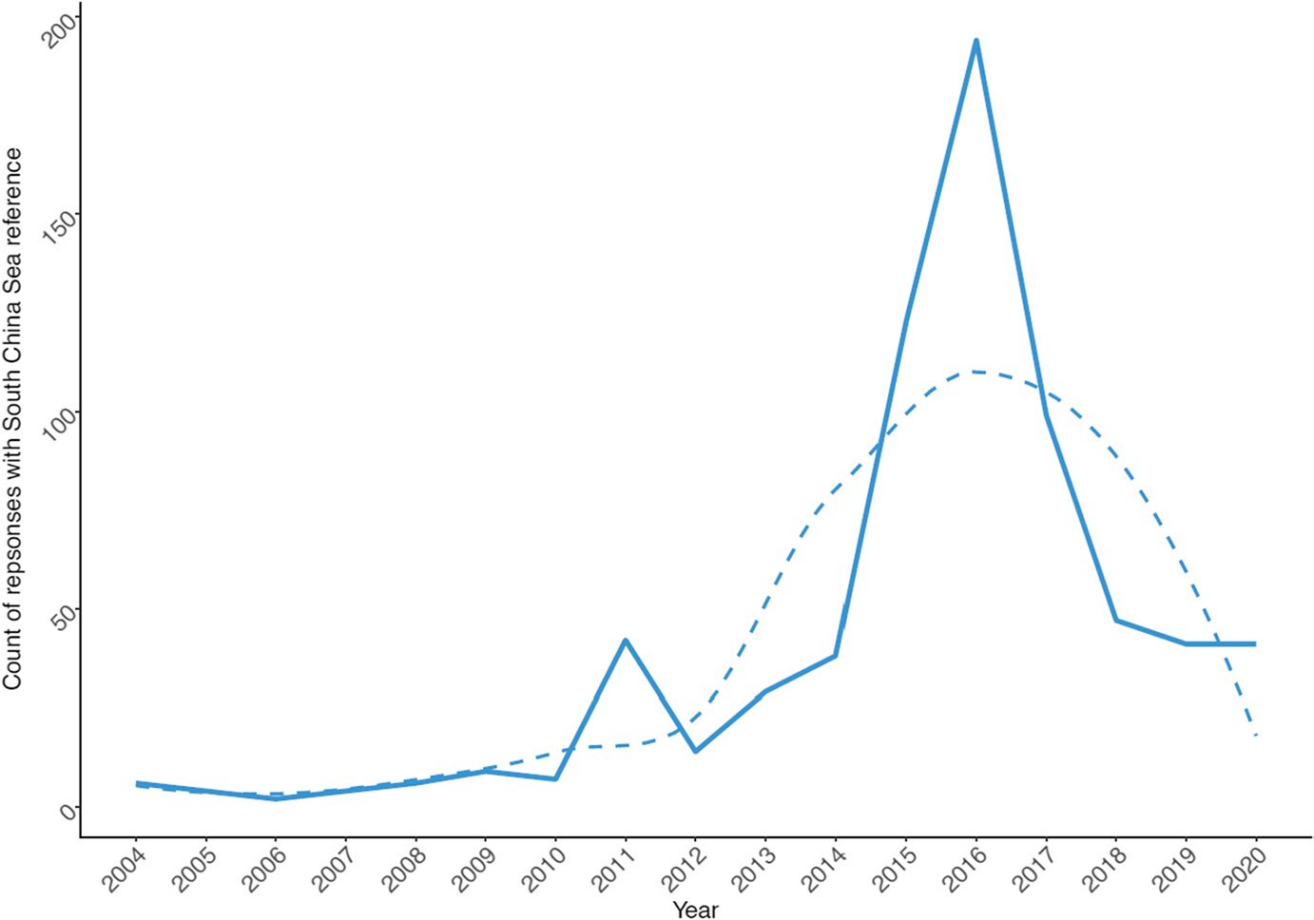
Number of answers concerning “South China Sea” per year. Note: Solid line visualizes raw counts. Dashed line visualizes smoothed trend (using loess function [Local Polynomial Regression Fitting])

Figure 7 visualizes the prominence of the reference to “South China Sea” in the Ministry’s answers over the covered period. We can notice that the number of mentions remained low during the 2000s, reaching the first minor peak only in 2011. Afterwards, the major peak was reached in 2016, when the decision of the arbitrary tribunal in The Hague was issued and which resulted in more than twice as many mentions of the South China Sea than in most other years [48]. Subsequently, the topic continued being discussed more often than even at the first minor peak of 2011.

Altogether, there were 706 questions and answers dealing with the South China Sea in our dataset. By far the most discussed claimant was the Philippines, appearing 181 times – far ahead of the second Vietnam which appeared 41 times. This reflects that especially the peak periods were driven by events involving the Philippines – such as the arbitration tribunal or the standoffs at the Scarborough and Second Thomas shoals. Interestingly, as a regional organization encompassing most of the claimants, ASEAN was mentioned only 22 times, showing that its involvement in the disputes has been relatively marginal. From the outside actors, the US was referred to 239 times – i.e. significantly more than any other actor. That may give credence to the claims that the South China Sea disputes are not “only” territorial disputes between regional countries but a playground for the broader US-China geopolitical struggle [13]. Japan too was a relatively important actor mentioned 51 time. No other actor appeared more than handful of times.

During the 2000s, South China Sea appeared in the discussion mainly after the actions of other countries – most of the times Vietnam and the Philippines – to which China’s response was solicited. The actions included attempts to exploit natural resources, building of an airport, opening a tourist route, high-profile visits to disputed territories, or administrative changes of status of the disputed territories. China’s responses to these actions were relatively similar: it claimed “*indisputable sovereignty over the islands and adjacent waters in South China Sea*”, suggested that the actions “*violate China’s sovereignty*”, and calling on the actors to cease their actions. It is noteworthy that China’s response remained relatively muted and even the requests were often framed as that China “hopes” that the actors would change their behaviour. Besides, the situation in the South China Sea was described overall as “*stable*” and China made it clear that while insisting on its sovereignty, it was also willing to “*lay aside differences and seek joint exploration*” until the final resolution of the disputes.

In 2009, Chinese own actions started to be discussed, such as increasing fishery activity, sea patrols, military presence, development of tourism, or submission of claims to the UN. While the tone of remarks targeting the neighbouring countries remained diplomatic, the spokespersons also started emphasizing China’s strong will to stand its ground, such as that “*the resolve of the Chinese Government to safeguard territorial integrity and maritime rights and interests is resolute*.” When the US was mentioned, however, the tone of the spokesperson’s remarks took quite different shape compared to the regional countries. The US’ claims are called as “*flatly inaccurate and unacceptable*”, “*sheer lies*”, and breaking international law. This development can be seen as the first signs of “Chinese assertiveness”, which also started to be discussed at about the same time and primarily in relation to the South China Sea disputes [50].

Post-2011 discussion on the South China Sea has increased significantly in quantity and China’s positions have hardened, as it has increased its activity (including massive build-up of artificial islands or putting pressure on other claimants – such as at the Scarborough Shoal or the Second Thomas Shoal), but also as a result of steps of other countries (the arbitration ruling initiated by the Philippines or various types of sea patrols and demonstrative maritime presence by other countries, particularly the US, but also Japan). At the same time, there has been a lot of continuity with the pre-2012 statements. Even when tensions were high between China and the Philippines (under Aquino III administration) or Vietnam (to a lesser extent), China’s rhetoric emphasized cooperation: “*China’s bilateral communication channels with the Philippines, Vietnam and other relevant countries on the South China Sea issue have been smooth and open*”. At the same time, China did not shy away from criticizing the nature of steps of other claimants, most clearly visible at the end of Aquino administration when the spokesperson had this comment: “*Dressed up as a victim, the Philippines keeps acting provocatively, stirring up troubles, aggravating tensions, and undermining regional peace and stability. China will never bully small countries, but we will in no way tolerate small country making up excuses and hurting China’s interests.*”

Discussing the role of non-claimants, China’s rhetoric would change, most significantly in relation to the US. Various statements of US actors are rejected as “*untrue and irresponsible remarks*”, or turning “*a blind eye to facts, confuse right and wrong*.” The role of the US – which is consistently called as a “non-party”, “foreign intervention”, or “outside forces” – is presented as singularly negative, complicating the otherwise “generally stable” situation in South China Sea. The US is said to “*play up*” and “*hype*” the issue which reflects its “*ulterior motives*”. Overall, “*wrong words and actions made by the US side on maritime issues have emboldened some countries to take provocative actions*.” At times, the criticism of the US gets personal: during 2020, former US State Secretary Mike Pompeo started to be referred to without any prefix title and went by simply as “Pompeo”, in an unusual step for the Ministry, which showed the level of negativity in the US-China relations in general and with the former secretary Pompeo in particular.

Other non-claimants got somewhat better treatment than the US (and much less space). Still, Japan was reminded to “*learn from history*” of the World War II during which it occupied islands in the South China Sea. Australia was told that it is “*not a party to the South China Sea dispute. It should base its position on the right and wrong of the matter, stick to its promise of not taking sides on disputes over territorial sovereignty, watch its words and actions, and avoid undermining regional peace and stability and bilateral relations*.”

Essentially, China’s message for the claimants is not to confront China and not to bring in outside powers or multilateral institutions – and in return be rewarded with regional peace and stability, as well as positive bilateral relations and cooperation with China. To the non-claimants, China paints them as having no right to get involved in South China Sea, where China and the regional countries are allegedly perfectly capable of preserving peace and stability, while any outside intervention would only make things worse.

## Conclusion

The paper presents an original corpus mapping the official Chinese foreign policy discourse over almost two decades during which China has become economic and political challenger of the western global dominance and the US in particular. The dataset is a unique source of information on official positions and diplomatic narratives of China over time. The presented case studies of US-related and South China Sea-related discourses were primarily meant to take two well-known areas of China’s international relations and illustrate how corpus-based approach (both quantitative and qualitative) may help to understand Chinese diplomatic positions and its evolution while maintaining a high level of reliability and external validity of presented arguments. We hope that collected corpus would become a standard reference source for scholars studying China both qualitatively and quantitatively.

We see further applications especially when it comes to mapping and tracking topics (counting and weighting of n-grams; topic-modelling), scaling of data (Wordfish; Wordscores), understanding the contexts in which political concepts occur (keywords in context; latent semantic analysis; word embeddings), studying relations among actors, organizations, and places (named entity recognition; relational mapping), detecting sentiments and emotions (sentiment analysis; emotion mining; opinion mining), and extracting information (argumentation mining; phrases extraction). The applications, however, are not limited to computer-assisted analyses. Traditional discourse analysis, close reading, and deep understanding of studied topics cannot be replaced by computer-ran algorithms, however, they can greatly benefit from the rigor and scale the corpus-based approaches can facilitate.

## Footnotes

1 Corpus and all supporting materials are available via Harvard Dataverse platform at 10.7910/DVN/BAKGET.

2 The conferences are transcribed and published online on the official website of the Ministry in Chinese, Arabic, English, French, Russian, and Spanish languages [11].

3 See the recent work of Dai and Luqiu [14] using Chinese transcripts of press conferences.

4 This claim is possible due the nature of the MFA website which at any given time stores approximately two years of press conferences. Dense snapshots taken by the Wayback Machine therefore hugely overlaps so any corrupt link in a snapshot of the website is corrected using a different one covering the same window. First such snapshot exists from 7 October, 2003 and covers press conferences back to 15 October 2002. Beyond that date, it is not possible to collect any additional data on press conferences using the Wayback Machine archive.

5 Over the studied period, eleven spokespersons held the press conferences: Geng Shuang (3382 responses), Hong Lei (2142 responses), Hua Chunying (4237 responses), Jiang Yu (1793 responses), Kong Quan (1294 responses), Liu Jianchao (2055 responses), Liu Weimin (240 responses), Lu Kang (2151 responses), Ma Zhaoxu (496 responses), Qin Gang (2440 responses), Wang Wenbin (581 responses), Zhang Qiyue (814 responses). 105 responses do not have any name assigned.
